# Association between body roundness index and risks of all-cause and cardiovascular mortality in adults with metabolic dysfunction-associated steatotic liver disease: NHANES 1999–2018

**DOI:** 10.3389/fnut.2025.1604398

**Published:** 2025-07-08

**Authors:** Yanshan Yi, Li Yang

**Affiliations:** Department of Occupational Health and Environmental Health, School of Public Health, Guangxi Medical University, Nanning, China

**Keywords:** metabolic dysfunction-associated steatotic liver disease, body roundness index, all-cause mortality, cardiovascular disease mortality, NHANES

## Abstract

**Background:**

Visceral obesity is an important risk factor for the development and progression of metabolic dysfunction-associated steatotic liver disease (MASLD). The body roundness index (BRI) is a novel indicator that demonstrates a stronger correlation with visceral fat than other anthropometric indices. However, the association between the BRI and mortality risk in patients with MASLD remains unclear. Therefore, this study investigated the relationship between the BRI and the risks of all-cause and cardiovascular disease mortality among patients with MASLD.

**Methods:**

This study included 7,428 adults aged ≥18 years with MASLD, utilizing data from the National Health and Nutrition Examination Survey (NHANES) database spanning from 1999 to 2018. The assessment of MASLD was conducted based on the fatty liver index (FLI). To examine the relationship between the BRI and mortality risks, multivariable Cox proportional hazards regression models, trend analysis, and restricted cubic spline curves were employed. Additionally, subgroup analyses were conducted to assess whether the association between the BRI and mortality varied across different subgroups.

**Results:**

In total, 1,249 participant deaths were recorded during a median follow-up period of 115 months, of which 404 were attributed to cardiovascular disease. After adjusting for multiple covariates in the fully adjusted model, the risk of all-cause mortality was increased by 27% (HR: 1.27; 95% CI: 1.00–1.60) and 52% (HR: 1.52; 95% CI: 1.18–1.96) in BRI quartiles 3 to 4 (Q3–Q4) compared with Q1, respectively. Similarly, the risk of cardiovascular disease mortality was increased by 61% (HR, 1.61; 95% CI, 1.05–2.46), 62% (HR, 1.62; 95% CI, 1.03–2.53), and 144% (HR, 2.44; 95% CI, 1.46–4.09) in BRI quartiles 2 to 4 (Q2–Q4) compared with Q1, respectively. The restricted cubic spline curves indicated a linear relationship between the BRI and both all-cause and cardiovascular disease mortality (*p* for non-linearity >0.05).

**Conclusion:**

In this nationally representative sample of adults with MASLD from the non-institutionalized civilian population in the United States, the BRI served as an independent predictor of both all-cause and cardiovascular disease mortality. Specifically, higher BRI values were associated with increased risks of both all-cause and cardiovascular disease mortality among patients with MASLD.

## 1 Introduction

Metabolic dysfunction-associated steatotic liver disease (MASLD) is a new term proposed by three major international liver associations in 2023, replacing the previous term “non-alcoholic fatty liver disease” and offering a redefined perspective based on non-alcoholic fatty liver disease (1–3). The nomenclature and definition of MASLD more accurately capture the pathophysiological and cardiometabolic implications of this prevalent liver disease (4). MASLD is a liver disease accompanied by systemic metabolic disorders (4). Its definition encompasses the presence of hepatic steatosis in the absence of excessive alcohol consumption, and it is accompanied by at least one cardiometabolic risk factor (including overweight, type 2 diabetes mellitus, hypertension, hypertriglyceridemia, or a low high-density lipoprotein cholesterol level) (1–3). MASLD is the most prevalent chronic liver disease globally (4). In recent years, its prevalence has been increasing alongside the rising incidence of obesity and diabetes (5). Currently, more than one-third of adults worldwide are affected by MASLD (6). Among the obese population, its prevalence is close to 70% (7). Without intervention, patients with MASLD are at risk of progressing to liver fibrosis, cirrhosis, and hepatocellular carcinoma (8, 9). Furthermore, MASLD is significantly associated with cardiovascular mortality and systemic complications such as chronic kidney disease, posing a major challenge to global public health (10).

Several studies have demonstrated that patients with MASLD have a higher risk of all-cause mortality than do individuals without fatty liver disease (11, 12). Cardiovascular death is the primary cause of mortality in patients with MASLD (13). Obesity is a crucial risk factor for an adverse prognosis in individuals with MASLD (14). Moreover, bariatric surgery, particularly laparoscopic procedures, has been shown to reduce mortality risk in these patients (15). Growing evidence indicates that visceral fat and subcutaneous fat exert different degrees of influence on the development of MASLD, with visceral fat seemingly having a more detrimental impact (6, 16). Visceral fat is associated with greater insulin resistance and cardiometabolic risks, significantly affecting the occurrence and progression of MASLD (17, 18). However, the most commonly used indicator of obesity, the body mass index (BMI), has been shown in a growing number of studies to have limitations because it does not account for the fat distribution across different body regions and thus cannot accurately assess different types of body weight (19, 20). Thomas et al. (21) proposed the body roundness index (BRI) as a new anthropometric measure, modeled on the shape of the human body and calculated from height, waist circumference, and body circumference. A previous study showed that the BRI is more strongly associated with visceral obesity than are traditional measures such as the BMI and other body metrics, including the body shape index, waist circumference, and waist-to-hip ratio (22). Additionally, the BRI is an independent predictor of all-cause mortality and cardiovascular mortality, a finding validated in the general population as well as in patients with various conditions such as diabetes and hypertension (23–25).

Nevertheless, the association between the BRI and mortality risk in patients with MASLD remains unclear. To address this research gap, we performed an analysis of data from the National Health and Nutrition Examination Survey (NHANES) to explore the association of the BRI with all-cause mortality risk and cardiovascular disease mortality risk among adults with MASLD.

## 2 Materials and methods

### 2.1 Study design and population

The National Health and Nutrition Examination Survey (NHANES) is a national cross-sectional and longitudinal research project sponsored by the Centers for Disease Control and Prevention to assess the health and nutritional status of the non-institutionalized civilian population in the United States. NHANES data are obtained through a stratified multistage probability sampling design to ensure a nationally representative sample. Since 1999, the NHANES database has been continuously updated on a 2-year cycle. The data for this study were extracted from the NHANES 1999–2018 cycles, spanning 10 survey cycles, and included demographics, dietary data, physical examination results, questionnaire responses, and laboratory data. The Research Ethics Review Board of the National Center for Health Statistics granted approval for the NHANES research protocol. During the enrolment process, all participants provided informed written consent.

**Figure 1:**
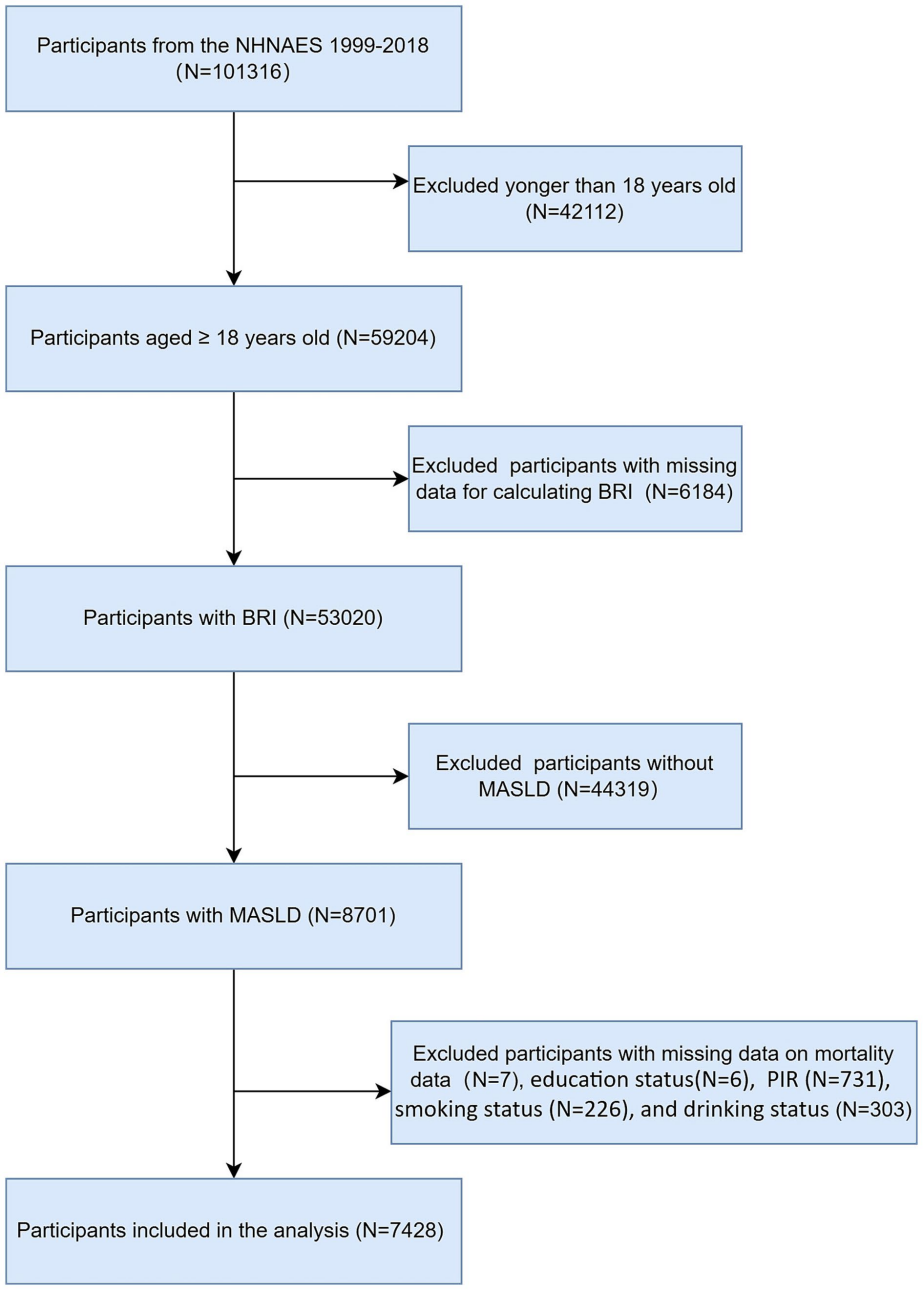
Flow chart of participants selection.

The NHANES dataset spanning from 1999 to 2018 encompassed a total of 101,326 participants. Initially, we excluded 42,112 individuals who were younger than 18 years of age from our analysis. We subsequently excluded participants with missing BRI data (*n* = 6,184) and further excluded those without MASLD (*n* = 44,319). Finally, we excluded participants with missing survival data and covariates (*n* = 1,273), ultimately including 7,428 participants in our study. The flowchart of participant selection is shown in Figure 1.

### 2.2 Definition of MASLD

MASLD is defined as a steatotic liver disease accompanied by at least one cardiometabolic risk factor, excluding individuals with viral hepatitis and those with alcohol intake of ≥30 g/day for men and ≥20 g/day for women (3). Given the absence of direct ultrasonographic assessment data for hepatic steatosis in most interview cycles of the NHANES dataset spanning from 1999 to 2018, this study utilized the fatty liver index (FLI) as a surrogate measure to evaluate steatotic liver disease. The FLI is a credible instrument for assessing fatty liver and is distinguished by its high sensitivity and specificity (26, 27). Previous studies have indicated that among various non-invasive tests, the FLI exhibits the highest area under the curve (AUC) value and the best ability in predicting MASLD (28). The FLI is calculated as follows (29):


$$\mathrm{FLI}=\frac{\left(e^{0.953\times \mathrm{Ln}(\mathrm{TG})+0.139\times \mathrm{BMI}+0.718\times \mathrm{Ln}(\mathrm{GGT})+0.053\times \mathrm{WC}-15.745}\right)}{\left(1+e^{0.953\times \mathrm{Ln}(\mathrm{TG})+0.139\times \mathrm{BMI}+0.718\times \mathrm{Ln}(\mathrm{GGT})+0.053\times \mathrm{WC}-15.745}\right)}\times 100$$

In this formula, TG stands for triglycerides, GGT for gamma-glutamyl transferase, BMI for body mass index, and WC for waist circumference. Previous studies have demonstrated that an FLI of ≥60 is associated with a high probability of hepatic steatosis (26, 29). Consequently, participants with an FLI of ≥60 were identified as having steatotic liver disease in this study. The cardiometabolic risk factors included (3) a BMI of ≥25 kg/m^2^ or waist circumference of ≥94 cm for men and ≥80 cm for women; fasting plasma glucose of ≥100 mg/dL, 2-h post-load plasma glucose of ≥140 mg/dL, glycated hemoglobin of ≥5.7%, or a diagnosis of diabetes mellitus or ongoing diabetes treatment with hypoglycemic agents; blood pressure of ≥130/85 mmHg or ongoing antihypertensive therapy; fasting plasma triglycerides of ≥150 mg/dL or ongoing lipid-lowering therapy; and plasma high-density lipoprotein cholesterol of <40 mg/dL for men and <50 mg/dL for women or ongoing lipid-lowering therapy.

### 2.3 Covariates

The covariates were age, sex, race, education, poverty income ratio (PIR), smoking status, alcohol consumption, and history of hypertension, diabetes, and cardiovascular disease. Age, sex, race, education, PIR, and smoking status were collected via questionnaires administered during household interviews. Study subjects whose ages surpassed the top-censor cutoff age in the NHANES survey were assigned the top-censor cutoff age. Alcohol consumption was obtained from the 24-h dietary recall interviews of the dietary questionnaire. The race categories were Mexican American, Non-Hispanic White, Non-Hispanic Black, and other race. Educational levels were classified as below high school, high school diploma, and above high school. Smoking status was categorized into never smoking, former smoking, and current smoking. Never smoking referred to individuals who had smoked no more than 100 cigarettes in their lifetime. Former smoking referred to individuals who had smoked more than 100 cigarettes in their lifetime but did not currently smoke. Current smoking referred to individuals who had smoked more than 100 cigarettes in their lifetime and continued to smoke. Hypertension was defined as having a systolic blood pressure of ≥140 mmHg, having a diastolic blood pressure of ≥90 mmHg, being on antihypertensive medication, or self-reporting a physician-diagnosed hypertension. Diabetes mellitus was defined as having a glycated hemoglobin level of ≥6.5%, a fasting plasma glucose level of ≥126 mg/dL, or a 2-h plasma glucose level of ≥11.1 mmol/L during an oral glucose tolerance test; being on antidiabetic medication or insulin therapy; or self-reporting a physician-diagnosed diabetes. Cardiovascular disease was defined as self-reporting a physician-diagnosed condition of congestive heart failure, coronary heart disease, angina pectoris, myocardial infarction, or stroke.

### 2.4 Definition of BRI

The following formula (21) was used to calculate the BRI: BRI = 364.2–365.5 × √[1 − (waist circumference in centimeters / 2π)^2^/(0.5 × height in meters)^2^]. The participants’ height and waist circumference were measured at the mobile examination center.

### 2.5 Outcomes

The outcomes of this study were all-cause mortality and cardiovascular disease mortality. We utilized mortality data (NHANES Public-Use Linked Mortality File) published on the Centers for Disease Control and Prevention website.^1^ Cardiovascular mortality was defined as death caused by heart diseases (ICD-10 codes I00-I09, I11, I13, and I20-I51) or cerebrovascular diseases (ICD-10 codes I60–I69). Follow-up time was calculated from the date participants underwent the examination, with the cutoff date set as 31 December 2019. Causes of death were defined according to the International Classification of Diseases, Tenth Revision codes.

### 2.6 Statistical analysis

Given the complex sampling design employed by the NHANES, all analyses in this study were conducted using exam sample weights. The BRI was divided into four quartiles, and the baseline characteristics of the participants within each quartile were described. Continuous variables are represented by means and standard error with comparisons between different groups conducted using analysis of variance. Categorical variables are presented as case numbers and percentages, with comparisons between groups conducted using the chi-square test. Kaplan–Meier curves were constructed, and the log-rank test was employed to compare survival rates among different quartiles of the BRI. Three Cox proportional hazards regression models were constructed to evaluate the associations between the BRI and all-cause mortality as well as cardiovascular disease mortality, with the BRI divided into quartiles as categorical variables and the lowest quartile serving as a reference. Model 1 was unadjusted. Model 2 was adjusted for demographic information, including age and sex. Model 3 was further adjusted for age, sex, race, education, PIR, smoking status, alcohol consumption, and history of diabetes, hypertension, and cardiovascular disease. In addition, for all Cox regression models, the proportional hazards assumption was assessed using the Grambsch–Therneau test. Trend analyses were conducted using the median of each BRI quartile as a continuous variable in the model. Restricted cubic spline curves were employed to analyze the nonlinear relationship between the BRI and both all-cause and cardiovascular disease mortality. Subgroup analyses were conducted to explore whether the relationship between the BRI and mortality risk varied across subgroups, and interaction effect tests were performed. Sensitivity analyses were conducted to evaluate the robustness of the associations between the BRI and mortality risks by excluding participants who died within 2 years of follow-up, excluding accidental deaths, and excluding participants from the first three cycles (NHANES 1999–2004). All analyses were performed using R software (version 4.4.2). *p*-values were two-sided, and *p* < 0.05 was considered statistically significant.

## 3 Results

### 3.1 Baseline characteristics

Among 7,428 participants, 53.0% (weighted) were male, and the mean age was 49.55 years. The mean BRI of the participants was 7.18. The baseline characteristics of the participants according to BRI quartiles are shown in Table 1. Compared with participants in the lowest quartile, those with higher BRIs were more likely to be older, female, and Non-Hispanic Black and have a history of hypertension, diabetes, or cardiovascular disease (all *p* < 0.05). Additionally, participants with higher BRIs were less likely to be alcohol consumers or current smokers (all *p* < 0.05).

**Table 1:** Baseline characteristics of adults with MASLD according to quartiles of BRI.

Characteristic	Overall *n* = 7,428	Q1 (<5.62) *n* = 1,627	Q2 (5.63–6.78) *n* = 1,917	Q3 (6.79–8.23) *n* = 1,960	Q4 (>8.23) *n* = 1,924	*p-*value
Age, years, mean (SE)	49.55 (0.28)	46.39 (0.46)	49.99 (0.50)	51.36 (0.43)	50.44 (0.52)	<0.001
Age group, *n* (%)						<0.001
<45 years	2,703 (38.9)	725 (44.8)	640 (37.2)	647 (35.6)	691 (37.8)	
45–64 years	2,785 (41.6)	627 (43.3)	747 (42.6)	710 (40.2)	701 (40.3)	
≥65 years	1,940 (19.5)	275 (11.9)	530 (20.1)	603 (24.3)	532 (21.8)	
Sex, *n* (%)						<0.001
Male	3,742 (53.0)	1,298 (80.8)	1,050 (56.7)	792 (41.4)	602 (32.8)	
Female	3,686.0 (47.0)	329 (19.2)	867 (43.3)	1,168 (58.6)	1,322 (67.2)	
Race, *n* (%)						<0.001
Mexican American	1,597 (9.6)	368 (10.0)	462 (10.7)	402 (9.2)	365 (8.4)	
Non-Hispanic White	3,404 (70.0)	754 (71.3)	861 (69.6)	886 (69.1)	903 (69.9)	
Non-Hispanic Black	1,425 (10.2)	246 (7.0)	346 (9.4)	404 (11.6)	429 (12.7)	
Other race	1,002 (10.3)	259 (11.8)	248 (10.2)	268 (10.1)	227 (9.0)	
Education, *n* (%)						0.005
<High school	1,014 (7.0)	180 (5.0)	301 (8.2)	281 (7.7)	252 (7.1)	
Completed high school	1,181 (12.2)	231 (11.3)	291 (11.7)	327 (12.8)	332 (12.8)	
>High school	5,233 (80.8)	1,216 (83.8)	1,325 (80.1)	1,352 (79.4)	1,340 (80.1)	
PIR, *n* (%)						<0.001
≤1.30	2,430 (22.4)	451 (18.7)	583 (20.7)	650 (23.0)	746 (27.0)	
1.31–3.5	2,070 (39.0)	608 (47.5)	544 (39.4)	499 (37.9)	419 (31.1)	
>3.5	2,928 (38.6)	568 (33.8)	790 (40.0)	811 (39.1)	759 (41.9)	
Smoking status, *n* (%)						0.003
Never	3,956 (53.1)	826 (52.5)	999 (53.0)	1,079 (53.3)	1,052 (53.8)	
Former	2,185 (29.4)	443 (26)	604 (31.4)	575 (30.2)	563 (30.0)	
Current	1,287 (17.5)	358 (21.6)	314 (15.6)	306 (16.5)	309 (16.3)	
Drinking, *n* (%)	1,023 (15.3)	322 (21.3)	307 (18.0)	231 (12.6)	163 (9.4)	<0.001
Hypertension, *n* (%)	3,994 (50.8)	681 (39.7)	987 (48.0)	1,129 (54.8)	1,197 (60.6)	<0.001
Diabetes, *n* (%)	2,222 (24.7)	291 (12.0)	527 (21.4)	638 (28.5)	766 (37.0)	<0.001
CVD, *n* (%)	1,060 (11.9)	161 (8.3)	252 (11.0)	294 (12.4)	353 (16.0)	<0.001
BRI, mean (SE)	7.18 (0.04)	4.93 (0.02)	6.19 (0.01)	7.46 (0.01)	10.15 (0.06)	<0.001
Height, cm, mean (SE)	169.68 (0.17)	175.53 (0.31)	170.08 (0.30)	167.40 (0.36)	165.71 (0.31)	<0.001
Waist, cm, mean (SE)	113.02 (0.24)	101.24 (0.18)	107.58 (0.19)	114.39 (0.25)	128.89 (0.36)	<0.001

PIR, poverty income ratio; CVD, cardiovascular disease, BRI, body roundness index.

### 3.2 Kaplan–Meier survival analysis curves for all-cause and cardiovascular mortality according to BRI quartiles

**Figure 2:**
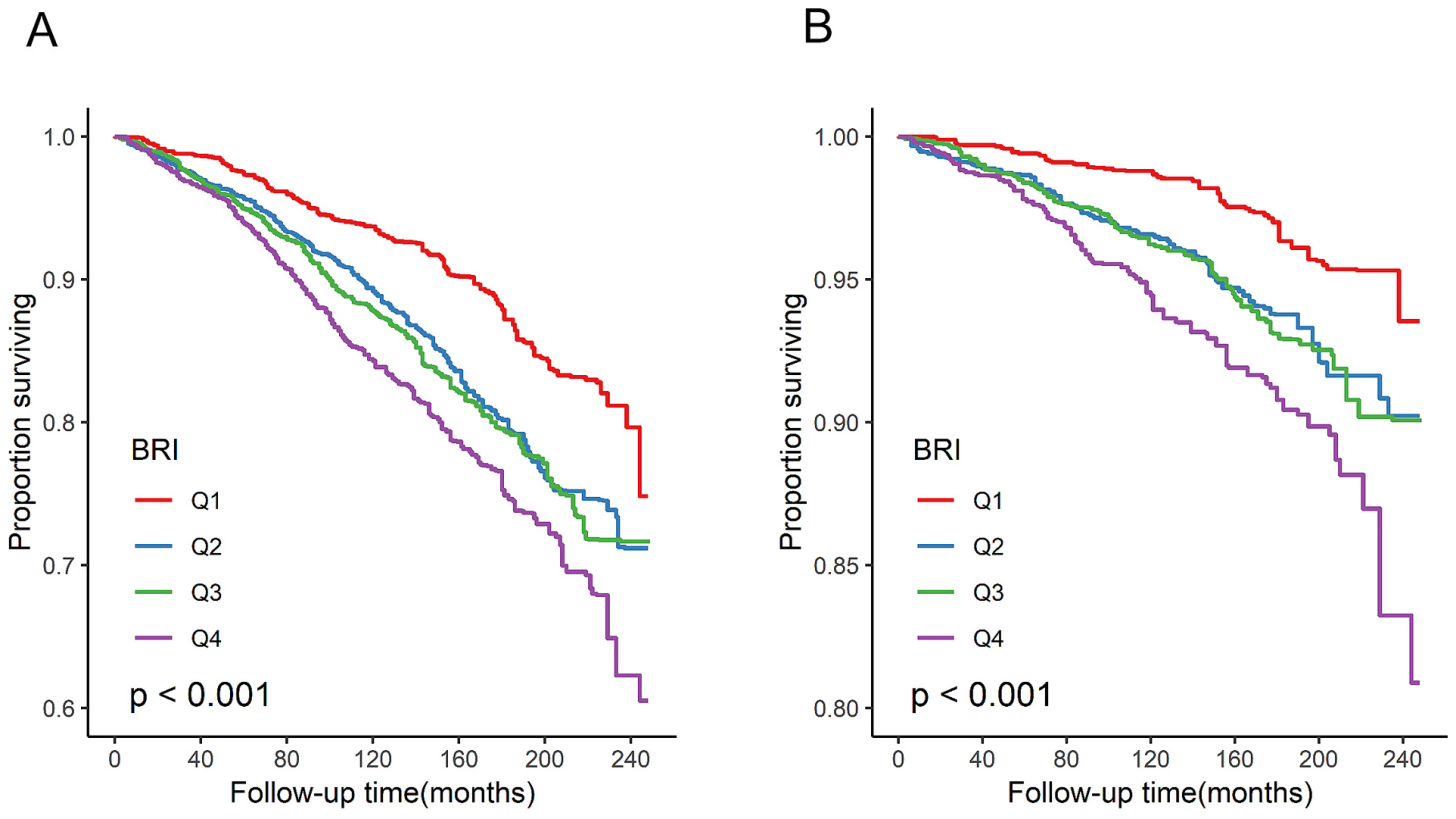
Kaplan–Meier survival analysis curves for all-cause and cardiovascular mortality. **(A)** All-cause mortality. **(B)** Cardiovascular disease mortality. All log-rank *p* < 0.001.

In total, 1,249 participant deaths were recorded during a mean follow-up period of 115 months, of which 404 were attributed to cardiovascular causes. Survival probability according to BRI quartiles is shown in Figure 2. Kaplan–Meier survival curves indicated significant differences in all-cause and cardiovascular mortality between BRI quartiles, with higher BRIs associated with lower survival probability (all log-rank *p* < 0.001).

### 3.3 Relationships of BRI with all-cause and cardiovascular mortality

In this study, three Cox proportional hazards regression models were developed to assess the association between the BRI and mortality risk. The relationship between the BRI and all-cause mortality as well as cardiovascular mortality is shown in Table 2. Among all included patients with MASLD, the BRI was positively associated with the risk of all-cause and cardiovascular disease mortality. After adjustment for multiple covariates (Model 3), the risk of all-cause mortality was increased by 27% (HR, 1.27; 95% CI, 1.00–1.60) and 52% (HR, 1.52; 95% CI, 1.18–1.96) in the BRI quartiles 3 to 4 (Q3–Q4) compared with the lowest quartile (Q1). Additionally, in the fully adjusted model (Model 3), the risk of cardiovascular mortality was increased by 61% (HR, 1.61; 95% CI, 1.05–2.46), 62% (HR, 1.62; 95% CI, 1.03–2.53), and 144% (HR, 2.44; 95% CI, 1.46–4.09) in BRI quartiles 2 to 4 (Q2–Q4) compared with the lowest quartile (Q1), respectively. In the fully adjusted model (Model 3), a significant linear relationship between the BRI and both all-cause and cardiovascular mortality was observed in the trend analysis (all *p* < 0.05).

**Table 2:** HRs (95% CIs) for all-cause mortality and CVD mortality according to BRI quartiles.

Regression model	Quartiles of BRI				*p* for trend
	Q1	Q2	Q3	Q4	
All-cause mortality					
Model 1 HR, (95% CI) *p*-value	1	1.61 (1.33, 1.95) < 0.001	1.72 (1.40, 2.13) < 0.001	2.18 (1.75, 2.71) < 0.001	<0.001
Model 2 HR, (95% CI) *p*-value	1	1.35 (1.10, 1.65) 0.004	1.45 (1.16, 1.83) 0.001	1.99 (1.58, 2.52) < 0.001	<0.001
Model 3 HR, (95% CI) *p*-value	1	1.22 (0.99, 1.49) 0.059	1.27 (1.00, 1.60) 0.046	1.52 (1.18, 1.96) 0.001	0.002
CVD mortality					
Model 1 HR, (95% CI) *p*-value	1	2.07 (1.38, 3.12) < 0.001	2.14 (1.42, 3.23) < 0.001	3.19 (2.03, 5.03) < 0.001	<0.001
Model 2 HR, (95% CI) *p*-value	1	1.78 (1.16, 2.72) 0.008	1.91 (1.25, 2.92) 0.003	3.26 (2.02, 5.26) < 0.001	<0.001
Model 3 HR, (95% CI) *p*-value	1	1.61 (1.05, 2.46) 0.027	1.62 (1.03, 2.53) 0.035	2.44 (1.46, 4.09) < 0.001	0.002

Model 1: non-adjusted; Model 2: adjusted for age and sex; Model 3: adjusted for age, sex, race, education, PIR, smoking, drinking, hypertension, diabetes, cardiovascular disease. HR, hazard ratio; CI, confidence interval. *p*-value of the Grambsch–Therneau test for all Cox regression models was >0.05.

### 3.4 Detection of nonlinear relationships

**Figure 3:**
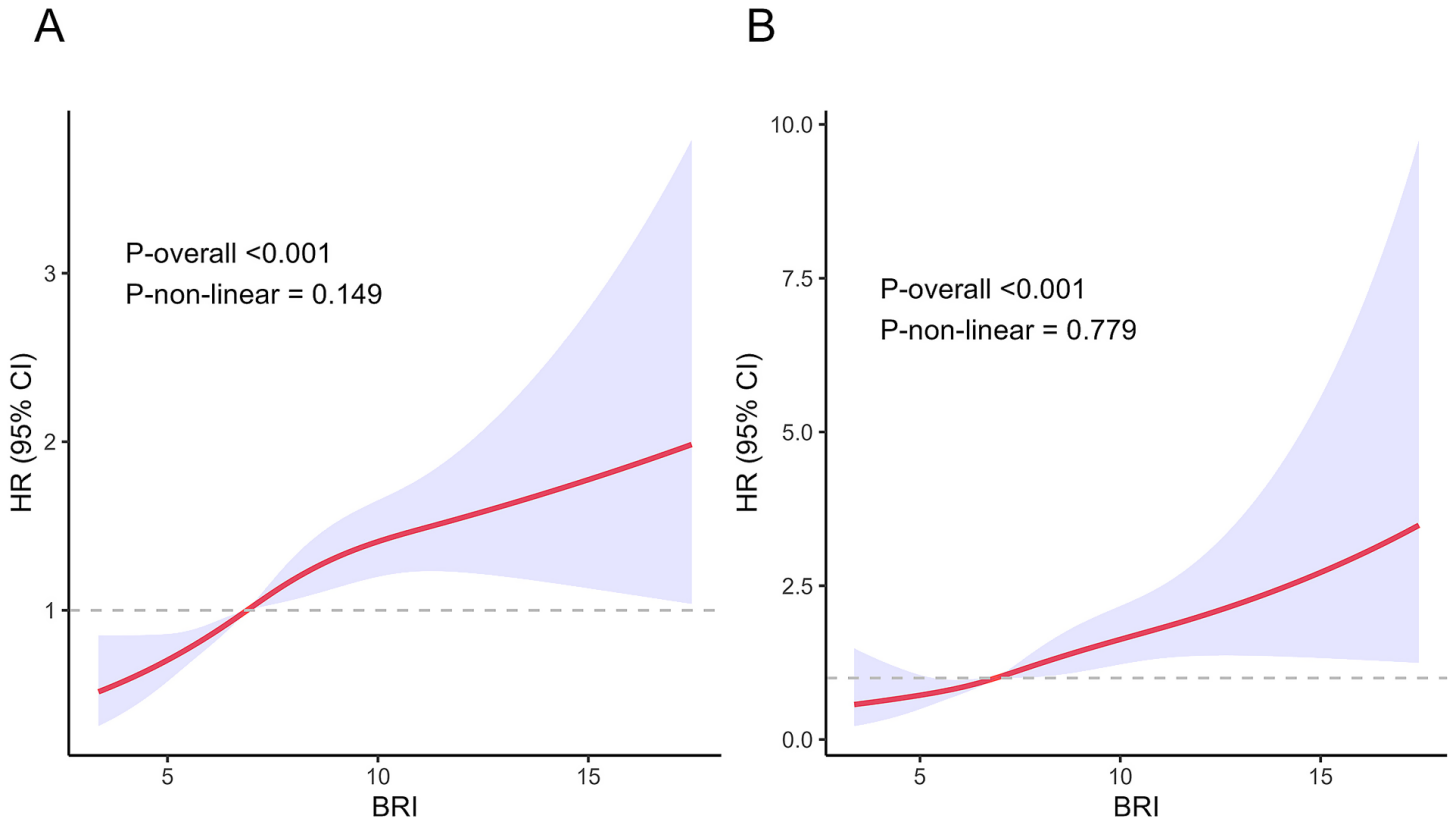
Association between BRI and all-cause and cardiovascular disease mortality in the MASLD population. **(A)** All-cause mortality. **(B)** Cardiovascular disease mortality.

The associations between the BRI and both all-cause and cardiovascular disease mortality exhibited approximately linear dose–response relationships (all *p* for non-linearity >0.05) (Figure 3). As the BRI increased, the risk of all-cause and cardiovascular disease mortality increased linearly.

### 3.5 Subgroup analyses

**Figure 4:**
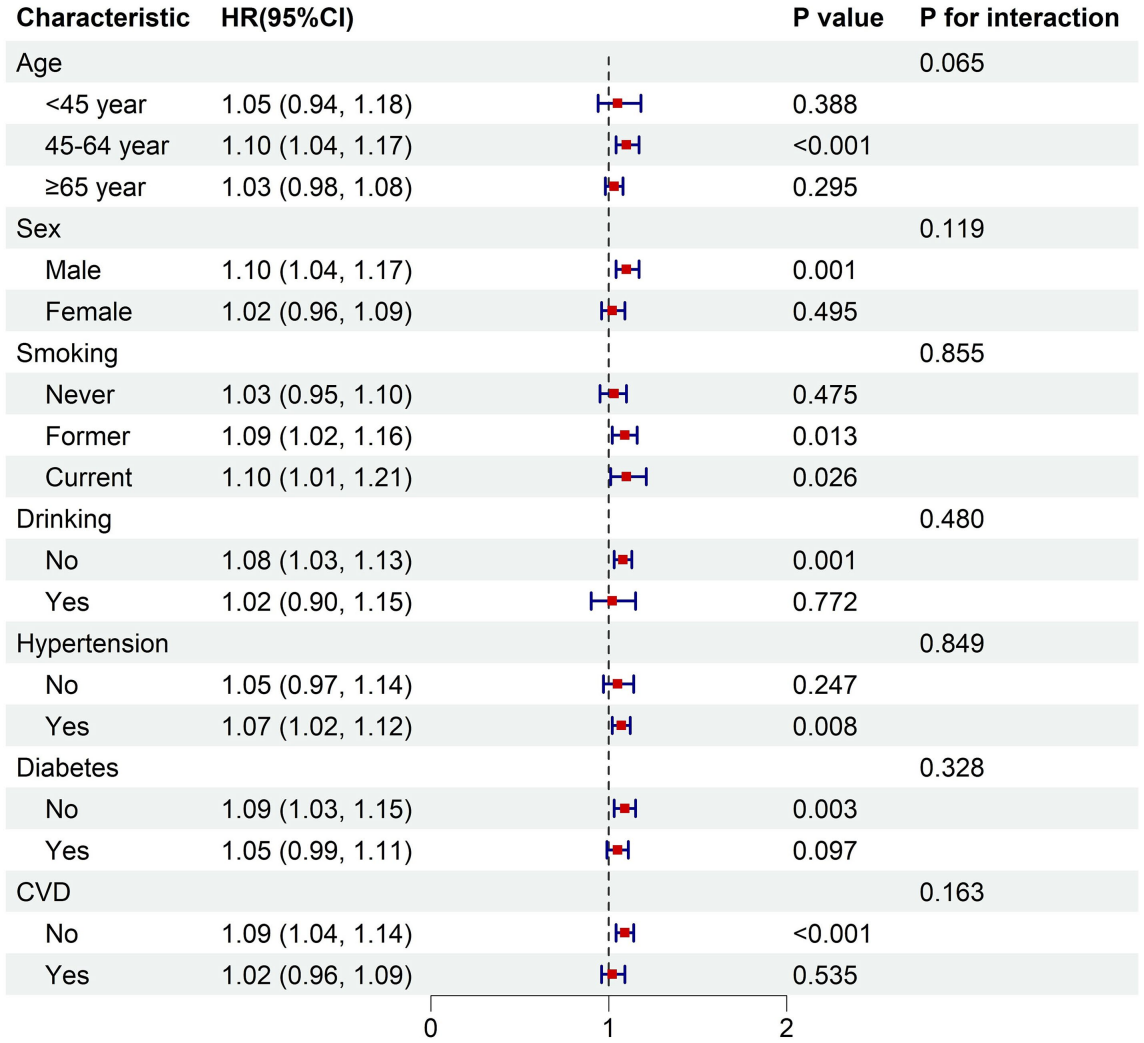
Subgroup analysis of the associations between BRI and all-cause mortality. The model was adjusted for age, sex, race, education, PIR, smoking status, drinking status, hypertension, diabetes, cardiovascular disease. PIR, poverty income ratio; CVD, cardiovascular disease; HR, hazard ratio for each one-unit increase in BRI.

**Figure 5:**
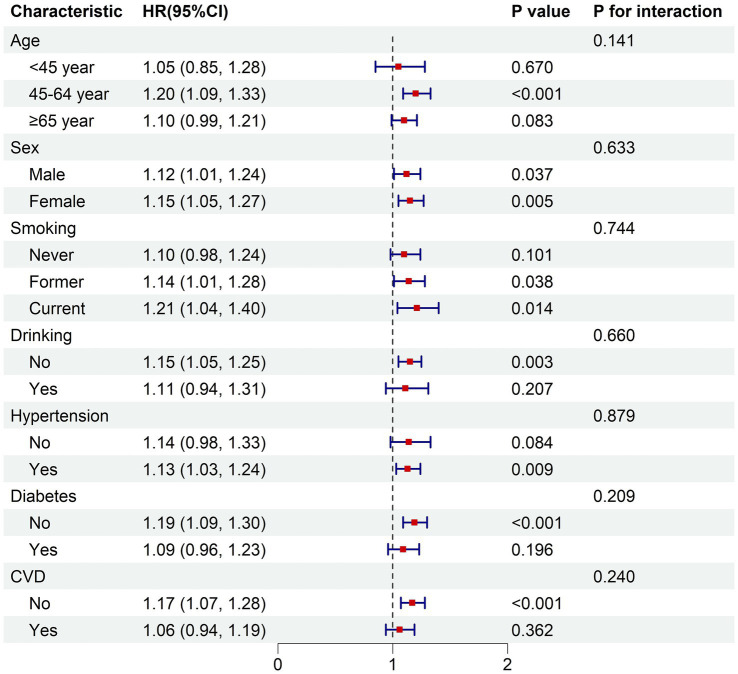
Subgroup analysis of the associations between BRI and cardiovascular mortality. The model was adjusted for age, sex, race, education, PIR, smoking status, drinking status, hypertension, diabetes, cardiovascular disease. PIR, poverty income ratio; CVD, cardiovascular disease; HR, hazard ratio for each one-unit increase in BRI.

In the fully adjusted model, the subgroup analysis demonstrated consistent relationships between the BRI and both all-cause mortality risk and cardiovascular disease mortality risk across various subgroups stratified by age, sex, smoking status, alcohol consumption, hypertension, diabetes, and cardiovascular disease (*p* for interaction >0.05) (Figures 4, 5).

### 3.6 Sensitivity analysis

To validate the robustness of the research results, we conducted sensitivity analyses by excluding participants who died within 2 years of follow-up, those who died accidentally, and participants from the NHANES 1999–2004 cycles (Supplementary Tables S1–S3). The results of these three sensitivity analyses demonstrated that the relationships between the BRI and both all-cause mortality risk and cardiovascular disease mortality risk were largely consistent with the primary analysis.

## 4 Discussion

To our knowledge, this is the first study to investigate the relationship between the BRI and the risks of all-cause mortality and cardiovascular disease mortality among individuals with MASLD. In this large-scale study utilizing nationally representative NHANES data, we demonstrated that patients with MASLD exhibiting higher BRIs have an elevated risk of all-cause mortality and cardiovascular disease mortality. Specifically, compared with patients who had MASLD with a BRI of <5.62, those with a BRI of >6.79 demonstrated a 27 to 52% increase in the risk of all-cause mortality and a 61 to 144% increase in the risk of cardiovascular disease mortality. These associations persisted after adjustment for relevant covariates, including demographic characteristics, physical examination indices, lifestyle habits, and comorbidities. Our findings indicate that the BRI possesses significant clinical value in predicting both all-cause mortality and cardiovascular disease-related mortality among patients with MASLD.

The BRI, a newly developed anthropometric index, demonstrates a strong correlation with visceral fat and offers practical advantages as a low-cost, non-invasive measure. Previous research has explored the association between the BRI and both all-cause mortality and cardiovascular disease mortality across diverse populations. A cohort study involving 32,995 American adults found a U-shaped relationship between the BRI and all-cause mortality (23); specifically, compared with adults who had BRI values in the middle quintile (4.5–5.5), those with BRIs of <3.4 had a 25% increased risk of all-cause mortality, while individuals with BRIs of ≥6.9 had a 49% increased risk (23). Similarly, a study among adults with diabetes or prediabetes in the United States reported a U-shaped association between the BRI and both all-cause and cardiovascular disease mortality, with thresholds of 5.54 and 5.21, respectively (24). When the BRI was below these thresholds, a negative correlation was observed between the BRI and all-cause mortality (HR, 0.87; 95% CI, 0.81–0.93), while the correlation with cardiovascular disease mortality was not significant (24); however, when the BRI exceeded these thresholds, a positive correlation emerged for both all-cause mortality (HR, 1.10; 95% CI, 1.06–1.14) and cardiovascular disease mortality (HR, 1.13; 95% CI, 1.07–1.20) (24). Similar findings have been reported in populations with hypertension, middle-aged and elderly individuals, and frail populations, further supporting the BRI as a valuable clinical predictor for both all-cause and cardiovascular disease mortality risk (25, 30, 31). These studies suggest that both excessively low and high BRI levels are linked to increased mortality risk. However, in contrast to these findings, our study demonstrated that among patients with MASLD, the BRI exhibited a linear rather than nonlinear dose–response relationship with all-cause and cardiovascular disease mortality risk. Specifically, our study showed that the risks of both all-cause and cardiovascular disease mortality increased as BRI levels rose. This may be attributed to the common coexistence of obesity in patients with MASLD. In addition, the FLI was used to define MASLD in this study, and the formula for the FLI, which includes BMI and waist circumference, overlaps with height and waist circumference in the formula for the BRI, which resulted in our selection of patients with MASLD who had an FLI ≥60 along with those who had a higher BRI. In our study, the average BRI in the lowest quintile was 4.93, which closely aligns with the middle quintile BRI (4.5–5.5) in the general population (23) and the thresholds reported in prior research (24, 25). This suggests that the BRI tends to be generally higher in patients with MASLD. This may explain the linear rather than nonlinear dose–response relationship between BRI levels and risk of mortality in patients with MASLD in the present study. Given that patients with MASLD typically have elevated BRI levels, it is reasonable to conclude that a higher BRI is associated with an increased risk of mortality in this population.

The biological mechanisms underlying the relationship between the BRI and mortality risk remain unclear. However, previous studies have suggested a potential link between visceral adipose tissue, metabolic disturbances, and adverse cardiovascular events. During the progression of obesity, excessive remodeling of visceral adipose tissue leads to adipocyte dysfunction (32). Dysfunctional visceral fat cells secrete pro-inflammatory and pro-fibrotic cytokines, which enter the portal vein, inducing inflammation and fibrosis in the liver as well as insulin resistance (33), thereby contributing to further systemic metabolic disturbances. Additionally, adipocytokines secreted by visceral fat, such as leptin and resistin, promote inflammation and endothelial dysfunction, leading to vasoconstriction and increasing the risk of vulnerability and rupture of atherosclerotic plaques (34, 35). Based on this, it can be inferred that the accumulation of visceral fat in patients with MASLD may exacerbate pre-existing metabolic disturbances, accelerate liver fibrosis, and increase the risks of adverse cardiovascular events and mortality.

In our study, a higher BRI was more commonly observed among older females and Non-Hispanic Black individuals. A nationwide study spanning from 1999 to 2018 identified an increasing trend in the BRI among American adults, with this growth being particularly pronounced among females, older adults, and Mexican Americans (23), which aligns with our findings. Additionally, patients with MASLD who had higher BRIs were less likely to be current smokers or alcohol consumers, potentially because of the higher proportion of females in this group. Furthermore, our study showed that patients with MASLD who had higher BRIs were more likely to have diabetes, hypertension, and cardiovascular disease. A longitudinal cohort study demonstrated a positive association between the BRI and hypertension risk, with each unit increase in the BRI being associated with a 17% increase in hypertension risk (36). Similarly, a cross-sectional study involving 11,980 adults older than 20 years indicated that the BRI can serve as a predictive indicator for diabetes and prediabetes, showing a positive correlation between the BRI and the incidence of both conditions (OR 1.17; 95% CI, 1.07–1.27) (37). Additionally, multiple studies have linked the BRI to cardiovascular disease risk, suggesting that the BRI can serve as a predictive factor for cardiovascular disease incidence (38, 39). These findings highlight that the BRI is correlated with various chronic diseases and represents a significant risk factor.

The analysis in this study is based on a nationally representative sample of patients with MASLD among adults from non-institutionalized civilian population in the United States and accounts for numerous confounding factors, enhancing the generalizability of the findings. However, this study has several limitations. First, as a cross-sectional study, it does not allow for causal inference. Second, because of the absence of ultrasound data, we utilized the FLI to define MASLD. Although the FLI has strong predictive capability for steatohepatitis, it serves primarily as a predictive tool for assessing MASLD, the lack of imaging data remains a limitation. Because of the overlap between the definitions of BRI and MASLD (both definitions include both height and waist circumference), this increases the possibility of collinearity, which may exaggerate the association between BRI and mortality outcomes. Finally, because the participants in this study were drawn exclusively from American adults, and because racial and ethnic differences in fat distribution or metabolic risk were not adequately accounted for in this study despite adjustments for race and other demographics, this limits the generalizability of the findings across diverse populations.

## 5 Conclusion

This study of a nationally representative sample from the non-institutionalized civilian population in United States showed that a dose–response relationship exists between the BRI and the risks of both all-cause mortality and cardiovascular disease mortality among patients with MASLD. Specifically, patients with higher BRI values exhibit an increased risk of both all-cause and cardiovascular disease mortality. Furthermore, the BRI serves as a strong independent predictor for both outcomes in patients with MASLD. As a simple and easily calculable indicator, routine clinical assessment of the BRI may aid in optimizing risk stratification and facilitating precise interventions for patients with MASLD.

## Data Availability

The original contributions presented in the study are included in the article/Supplementary material, further inquiries can be directed to the corresponding author.

## Footnotes

^1^ https://www.cdc.gov/nchs/data-linkage/mortality-public.htm
