# Supplementary material for: Association between body roundness index and risks of all-cause and cardiovascular mortality in adults with metabolic dysfunction-associated steatotic liver disease: NHANES 1999–2018
Source: Front Nutr. 2025 Jul 8;12:1604398. doi: 10.3389/fnut.2025.1604398 (PMC12279492; doi:10.3389/fnut.2025.1604398)
Supplement: Supplementary file 1 [file Table_1.docx]

Supplementary Material

**TABLE S1** HRs (95% CIs) for all-cause mortality and CVD mortality according to BRI quartiles after excluding deceased within two years of follow-up

| Regression model | Quartiles of BRI | | | |  | *P* for trend |
| --- | --- | --- | --- | --- | --- | --- |
|  | Q1 | Q2 | Q3 | Q4 |  |  |
| **All-cause mortality** | |  |  |  |  |  |
| Model 1  HR (95% CI) P value | 1 | 1.61(1.31, 1.97)<0.001 | 1.78(1.42, 2.23)<0.001 | 2.19(1.74, 2.76)<0.001 | | <0.001 |
| Model 2  HR (95% CI) P value | 1 | 1.35(1.09, 1.66)0.006 | 1.51(1.19, 1.93)<0.001 | 2.02(1.58, 2.59)<0.001 | | <0.001 |
| Model 3  HR (95% CI) P value | 1 | 1.21(0.99, 1.49)0.066 | 1.31(1.03, 1.68)0.029 | 1.53(1.17, 1.99)0.002 | | 0.003 |
| **CVD mortality** | |  |  |  |  |  |
| Model 1  HR (95% CI) P value | 1 | 1.82(1.20, 2.75)0.005 | 2.16(1.42, 3.27)<0.001 | 3.07(1.92, 4.90)<0.001 | | <0.001 |
| Model 2  HR (95% CI) P value | 1 | 1.55(1.02, 2.37)0.041 | 1.93(1.26, 2.97)0.003 | 3.15(1.92, 5.18)<0.001 | | <0.001 |
| Model 3  HR (95% CI) P value | 1 | 1.40(0.92, 2.13)0.110 | 1.62(1.03, 2.55)0.038 | 2.33(1.36, 3.98)0.002 | | 0.002 |

Model 1: Non-adjusted; Model 2: Adjusted for age and sex; Model 3: Adjusted for age, sex, race, education , PIR, smoking, drinking, hypertension, diabetes, cardiovascular disease. HR: Hazard ratio; CI: Confidence interval.The P-value of the Grambsch - Therneau test for all COX regression models was > 0.05.

**TABLE S2** HRs (95% CIs) for all-cause mortality and CVD mortality according to BRI quartiles after excluding participants with accidental deaths

| Regression model | Quartiles of BRI | | | |  |  |
| --- | --- | --- | --- | --- | --- | --- |
|  | Q1 | Q2 | Q3 | Q4 |  | *P* for trend |
| **All-cause mortality** |  |  |  |  |  |  |
| Model 1  HR (95% CI) P value | 1 | 1.67(1.37, 2.04)<0.001 | 1.82(1.45, 2.27)<0.001 | 2.29(1.82, 2.87)<0.001 | | <0.001 |
| Model 2  HR (95% CI) P value | 1 | 1.38(1.12, 1.71）0.003 | 1.51(1.19, 1.91）<0.001 | 2.07(1.62, 2.63）<0.001 | | <0.001 |
| Model 3  HR (95% CI) P value | 1 | 1.24(1.00, 1.54)0.045 | 1.31(1.03, 1.66)0.029 | 1.56(1.21, 2.01)<0.001 | | 0.001 |
| **CVD mortality** |  |  |  |  |  |  |
| Model 1  HR (95% CI) P value | 1 | 2.07(1.37, 3.11)<0.001 | 2.13(1.42, 3.21)<0.001 | 3.18(2.02, 5.01)<0.001 | | <0.001 |
| Model 2  HR (95% CI) P value | 1 | 1.77(1.16, 2.71）0.008 | 1.90(1.24, 2.91）0.003 | 3.23(2.00, 5.23）<0.001 | | <0.001 |
| Model 3  HR (95% CI) P value | 1 | 1.61(1.05, 2.45)0.028 | 1.61(1.03, 2.52)0.037 | 2.43(1.45, 4.06)<0.001 | | 0.002 |

Model 1: Non-adjusted; Model 2: Adjusted for age and sex; Model 3: Adjusted for age, sex, race, education , PIR, smoking, drinking, hypertension, diabetes, cardiovascular disease. HR: Hazard ratio; CI: Confidence interval.The P-value of the Grambsch - Therneau test for all COX regression models was > 0.05.

**TABLE S3** HRs (95% CIs) for all-cause mortality and CVD mortality according to BRI quartiles after excluding NHANES 1999-2004 participants

| Regression model | Quartiles of BRI | | | |  |
| --- | --- | --- | --- | --- | --- |
|  | Q1 | Q2 | Q3 | Q4 | *P* for trend |
| **All-cause mortality** | |  |  |  |  |
| Model 1  HR (95% CI) P value | 1 | 1.78(1.25, 2.53)0.001 | 2.03(1.45, 2.85)<0.001 | 2.49(1.71, 3.62)<0.001 | <0.001 |
| Model 2  HR (95% CI) P value | 1 | 1.39(0.97, 1.99)0.076 | 1.51(1.05, 2.17)0.026 | 2.12(1.45, 3.08)<0.001 | 0.004 |
| Model 3  HR (95% CI) P value | 1 | 1.25(0.85, 1.82)0.300 | 1.26(0.86, 1.84)0.200 | 1.60(1.05, 2.43)0.028 | 0.028 |
| **CVD mortality** |  |  |  |  |  |
| Model 1  HR (95% CI) P value | 1 | 3.95(2.04, 7.67)<0.001 | 3.32(1.66, 6.63)<0.001 | 5.57(2.76, 11.3)<0.001 | <0.001 |
| Model 2  HR (95% CI) P value | 1 | 3.08(1.53, 6.20)0.002 | 2.50(1.20, 5.22)0.015 | 5.05(2.41, 10.6)<0.001 | <0.001 |
| Model 3  HR (95% CI) P value | 1 | 2.98(1.42, 6.25)0.004 | 2.21(1.02, 4.81)0.045 | 4.18(1.91, 9.15)<0.001 | 0.007 |

Model 1: Non-adjusted; Model 2: Adjusted for age and sex; Model 3: Adjusted for age, sex, race, education , PIR, smoking, drinking, hypertension, diabetes, cardiovascular disease. HR: Hazard ratio; CI: Confidence interval.The P-value of the Grambsch - Therneau test for all COX regression models was > 0.05.
